# An In Vitro Study of the Anti-Acne Effects of *Scutellaria barbata*

**DOI:** 10.3390/molecules30030515

**Published:** 2025-01-23

**Authors:** Qiwen Zheng, Xiangji Jin, Trang Thi Minh Nguyen, Se-Jig Park, Gyeong-Seon Yi, Su-Jin Yang, Tae-Hoo Yi

**Affiliations:** 1 Graduate School of Biotechnology, Kyung Hee University, 1732 Deogyeong-aero, Giheung-gu, Yongin-si 17104, Republic of Korea; zhengqiwen@khu.ac.kr (Q.Z.); trangnguyen@khu.ac.kr (T.T.M.N.); tpwlt@khu.ac.kr (S.-J.P.); stella@khu.ac.kr (S.-J.Y.); 2 Department of Dermatology, School of Medicine, Graduate School, Kyung Hee University, 26 Kyungheedae-ro, Dong-daemun, Seoul 02447, Republic of Korea; hyanghe112@khu.ac.kr; 3 Department of Biopharmaceutical Biotechnology, Graduate School, Kyung Hee University, 1732 Deogyeong-daero, Giheung-gu, Yongin-si 17104, Republic of Korea; ks010924@khu.ac.kr

**Keywords:** *Scutellaria barbata*, anti-acne, anti-inflammatory, barrier protective

## Abstract

Acne is a common skin disease that is closely associated with *Cutibacterium acnes* (*C. acnes*) and the inflammatory response it induces. Existing antibiotic treatments are often rendered ineffective due to the development of bacterial resistance, while *Scutellaria barbata* (SLB) has attracted widespread attention for its remarkable anti-inflammatory and antibacterial properties. However, its role in acne treatment has not been comprehensively studied. This study used high-performance liquid chromatography (HPLC) to analyze the bioactive components in a 70% ethanol extract of SLB. The antibacterial activity against *C. acnes* was systematically evaluated using well diffusion, minimum inhibitory concentration (MIC), minimum bactericidal concentration (MBC), and biofilm formation assays. Additionally, the effects of SLB on nitric oxide (NO) production and phagocytic activity were tested in RAW 264.7 cells. An acne skin model was established by treating HaCaT keratinocytes with heat-inactivated *C. acnes*. The results demonstrated that SLB significantly inhibited the growth of *C. acnes* and disrupted its biofilm formation. Moreover, SLB markedly reduced the secretion of inflammatory cytokines such as interleukin (IL)-6, IL-1β, IL-8, and tumor necrosis factor (TNF)-α in HaCaT keratinocytes stimulated by *C. acnes*. Moreover, SLB effectively alleviated skin barrier damage caused by *C. acnes* by suppressing the expression of matrix metalloproteinases (MMPs)-1, -3, -9, and -13. In conclusion, this study demonstrates that SLB possesses potent antibacterial, anti-inflammatory, and barrier-protective properties, making it a promising candidate for developing anti-acne products and exploring alternative antibiotic therapies.

## 1. Introduction

Acne is a complex, multifactorial skin condition [1]. During its active phase, it often causes pain, skin damage, and changes in appearance, which can lead to reduced self-esteem, anxiety, depression, and social difficulties [2]. Furthermore, residual scars and hyperpigmentation after recovery significantly impact esthetics and mental well-being [3]. Recent studies have highlighted that the onset age of acne has expanded beyond the traditional adolescent period, with a notable increase in cases among both children and adults [4]. This shift poses new challenges and demands for the prevention and treatment of acne.

The core pathological basis of acne includes inflammation and immune response, sebaceous gland hyperactivity, abnormal follicular keratinization, and the overgrowth of *Cutibacterium acne* (*C. acne*) [5]. Among these, inflammation and immune responses play a key driving role [6]. *C. acne* activates inflammatory pathways in HaCaT keratinocytes, inducing the release of inflammatory cytokines such as interleukin (IL)-1β, IL-8, and tumor necrosis factor (TNF)-α, which attract macrophages to the affected area via chemotaxis [7]. This ultimately leads to follicular wall rupture and the development of inflammatory lesions. Additionally, *C. acne* stimulates HaCaT cells to secrete matrix metalloproteinases (MMPs), enzymes that break down components of the extracellular matrix (ECM), compromising the skin barrier [8]. This progression facilitates the transformation of acne from non-inflammatory lesions (e.g., comedones) to inflammatory lesions (e.g., pustules and cysts).

Given the pivotal roles of *C. acnes* and inflammation in acne pathogenesis, antibacterial and anti-inflammatory therapies have become key strategies for managing acne. However, current treatments face notable challenges. For instance, *C. acnes* can form biofilms at infection sites, reducing drug efficacy and promoting antibiotic resistance [9,10]. Furthermore, resistance to commonly used antibiotics such as clindamycin and tetracycline has steadily increased, further limiting their effectiveness and application [11]. In addition, 13-cis-retinoic acid, a widely used treatment, has been associated with adverse cardiovascular effects [12]. Natural alternatives like *C. obtusa* (Japanese cypress) extract and tea tree oil have been explored for their ability to reduce sebum production. However, the therapeutic efficacy of *C. obtusa* extract is suboptimal, and concerns over the toxicity of tea tree oil remain significant [13]. These challenges highlight the urgent need for alternative therapeutic strategies that are more effective and safer for managing acne.

*Scutellaria barbata* (SLB), a commonly used herb in traditional Chinese medicine, belongs to the Scutellaria genus of the Lamiaceae family. It is rich in bioactive compounds such as baicalin and baicalein, which possess strong antioxidant properties and are often employed in cancer treatments [14]. It has been found to possess significant activity in combating methicillin-resistant *Staphylococcus aureus* [15]. Furthermore, its active components exhibit anti-inflammatory effects in RAW264.7 cells [16]. However, there have been no reports on the anti-acne effects of SLB.

In this study, we investigated the inhibitory effects of SLB on *C. acnes* in vitro and its impact on *C. acnes*-induced inflammation in HaCaT keratinocytes. By demonstrating its antibacterial, anti-inflammatory, and skin barrier-protective properties, we aimed to explore the potential of SLB as a natural therapeutic agent for acne management.

## 2. Results

### 2.1. Chemical Contents of SLB

**Figure 1:**
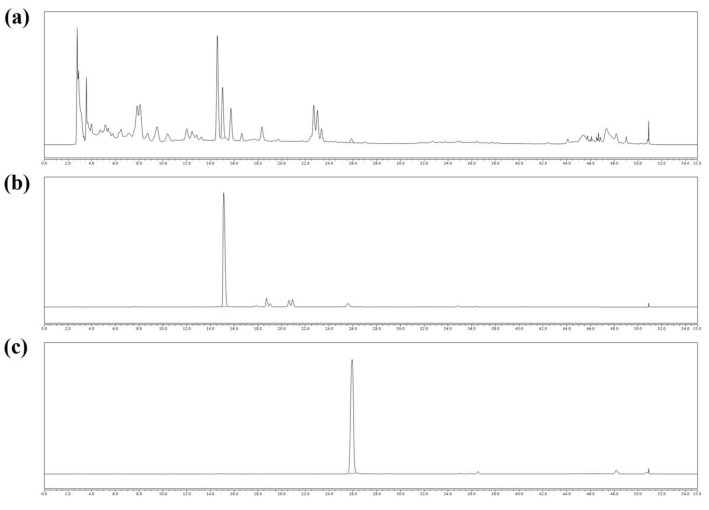
HPLC chromatograms of SLB (**a**), baicalin standards (**b**), and baicalein standards (**c**).

The contents of baicalin and baicalein detected in SLB extracts were 3.67% and 0.19%, respectively (Figure 1).

### 2.2. Antimicrobial Activity

**Figure 2:**
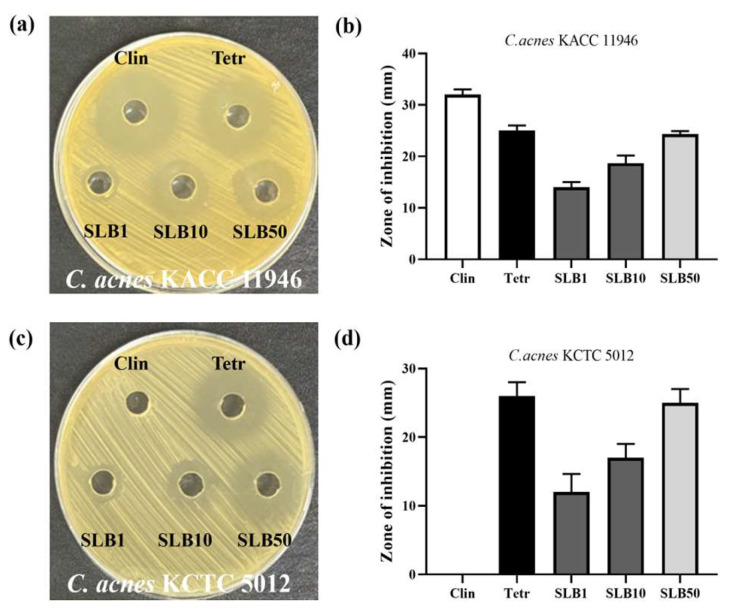
Antibacterial assay. Agar well diffusion showing the inhibition zones of SLB against *C. acnes KACC 11946*, with Clin and Tetr as the controls (**a**); the quantified zones of inhibition for the supernatant against *C. acnes KACC 11946* (**b**); agar well diffusion showing the inhibition zones of the supernatant and ethyl acetate fraction against *C. acnes KCTC 5012* (**c**); the quantified zones of inhibition for the supernatant and ethyl acetate fraction against *C. acnes KCTC 5012* (**d**). The presentation of the results involves the average ± SD from three independent experiments. Clin and Tetr refer to clindamycin (10 μg/mL) and tetracycline (10 μg/mL), respectively. SLB1, SLB10, and SLB50 represent SLB at concentrations of 1, 10, and 50 μg/mL, respectively.

The agar well diffusion method was employed to assess the antimicrobial effects of SLB on two *C. acnes* strains, KACC 11946 and KCTC 5012. A concentration of 50 μg/mL SLB exhibited an inhibition zone of 24 mm against KACC 11946, which is comparable to clindamycin (25 mm) (Figure 2a,b). This indicates that SLB is nearly as effective as clindamycin in inhibiting KACC 11946.

For KCTC 5012, 100 μg/mL SLB displayed an inhibition zone of 32 mm, whereas clindamycin and tetracycline showed zones of 0 mm and 26 mm, respectively (Figure 2c,d). Notably, the antimicrobial activities of clindamycin and tetracycline against these two *C. acnes* strains differed significantly. This suggests that KCTC 5012 may have developed resistance to clindamycin. Interestingly, SLB demonstrated consistent inhibitory effects against both *C. acnes* strains, highlighting its potential as a broad-spectrum agent against this pathogen.

### 2.3. Minimum Inhibitory Concentration (MIC) and Minimum Bactericidal Concentration (MBC) Assays

The MIC and MBC of SLB required to eliminate *C. acnes* KACC 11946 and *C. acnes* KCTC 5012 were determined. The findings revealed that the MBC of SLB required to effectively eliminate *C. acnes* strains KACC 11946 and KCTC 5012 was consistently greater than 0.16 mg/mL, as shown in Table 1. However, the MIC of SLB varied between the two strains, requiring concentrations above 0.04 mg/mL for *C. acnes* KACC 11946 and above 0.08 mg/mL for *C. acnes* KCTC 5012 (Table 1).

### 2.4. Morphological Alterations in C. acnes

**Figure 3:**
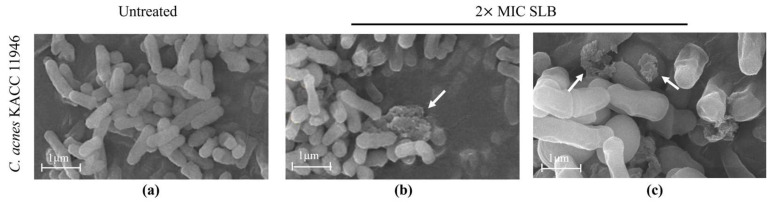
SEM of *C. acnes* KACC 11946 before and after treatment with SLB. Untreated control (**a**). Treated with SLB at 10,000× magnification (scale bar: 1 µm) (**b**). Treated with SLB at 50,000× magnification (scale bar: 1 µm) (**c**). White arrows illustrate cell wall breaking and fragments of *C. acnes* KACC 11946 after SLB treatment.

The cell wall, a sturdy layer surrounding the cell membrane, is essential for preserving cell shape and shielding against external factors. Morphological alterations in the bacterial cell wall of *C. acnes* KACC 11946 were analyzed using scanning electron microscopy (SEM) following SLB treatment. The untreated *C. acnes* KACC 11946 exhibited a smooth surface (Figure 3a), whereas SLB-treated *C. acnes* KACC 11946 showed severe cell wall disruption (Figure 3b,c).

### 2.5. Inhibition of Biofilm Formation

**Figure 4:**
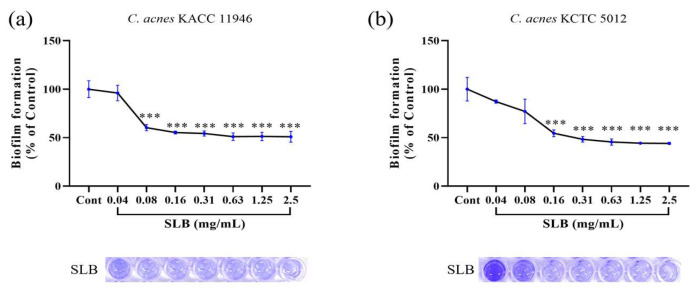
The antibiofilm formation activity of SLB against *C. acnes* KACC 11946 (**a**) and *C. acnes* KCTC 5012 (**b**). The presentation of the results involves the average ± SD from three independent experiments. *** *p* < 0.001 compared to the control group.

The inhibitory effects of SLB on biofilm formation by *C. acnes* strains were evaluated and are presented in Figure 4. SLB exhibited a dose-dependent inhibition of biofilm formation in both *C. acnes* KACC 11946 (Figure 4a) and *C. acnes* KCTC 5012 (Figure 4b). For *C. acnes* KACC 11946, the inhibitory activities at concentrations of 0.04, 0.08, 0.16, 0.31, 0.63, 1.25, and 2.50 mg/mL were 3.92%, 39.65%, 44.70%, 45.71%, 48.90%, and 49.08%, respectively. Similarly, for *C. acnes* KCTC 5012, SLB demonstrated inhibition rates of 12.75%, 22.94%, 45.46%, 51.73%, 54.49%, 55.66%, and 55.93% at the same concentrations. These findings indicate that SLB effectively inhibits biofilm formation in both strains, with greater inhibitory activity observed against *C. acnes* KCTC 5012.

### 2.6. Inhibitory Activity of Nitric Oxide (NO) Production

**Figure 5:**
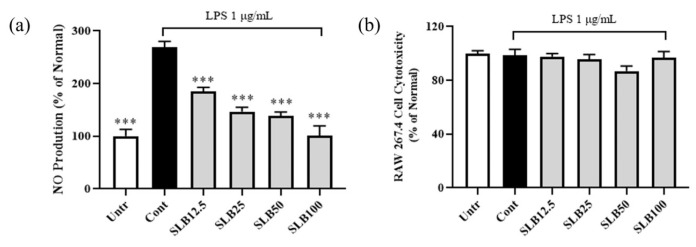
The inhibitory effect of SLB on LPS-induced NO production in RAW 264.7 cells (**a**) and the cytotoxicity of SLB (**b**). The presentation of the results involves the average ± SD from three independent experiments. *** *p* < 0.001 compared to the control group. SLB12.5, SLB25, SLB50, and SLB100 represent SLB at concentrations of 12.5, 25, 50, and 100 μg/mL, respectively.

During the development of acne, NO is considered an important mediator that accelerates the inflammatory response [17]. As shown in Figure 5a, NO synthesis in Lipopolysaccharide (LPS)-treated RAW 264. 7 cells significantly increased by 169.46% after 24 h compared to untreated cells. However, SLB treatment at different concentrations (12.5, 25, 50, and 100 µg/mL) significantly inhibited NO secretion compared to the LPS-treated control group, with inhibition rates of 31.46%, 45.53%, 48.77%, and 62.43%, respectively, exhibiting a dose-dependent decreasing trend.

To rule out the possibility that the reduction in NO levels was due to cytotoxicity, we measured cell viability in LPS- and SLB-treated cells using the MTT assay. As shown in Figure 5b, neither SLB nor LPS treatment significantly affected the viability of RAW 264.7 cells compared to the normal group. These findings indicate that the inhibitory effect of SLB on NO secretion is not caused by cell death but rather through its effective anti-inflammatory mechanism.

### 2.7. Effects of SLB on Phagocytosis

**Figure 6:**
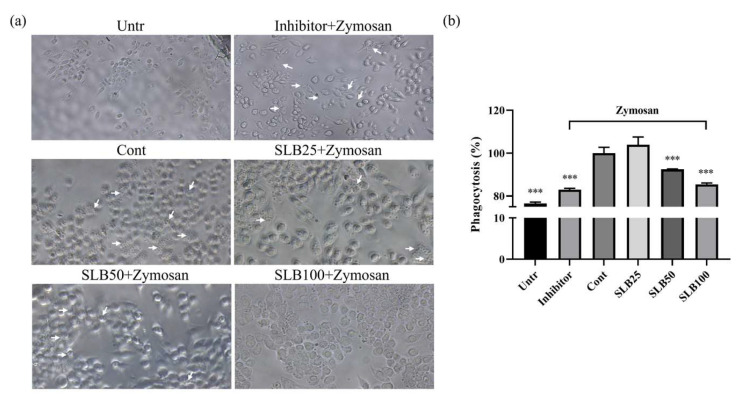
SLB enhances the phagocytic function of RAW 264.7 cells. Microscopic images (white arrows illustrate the location of Zymosan) (**a**) and ELISA results (**b**). The presentation of the results involves the average ± SD from three independent experiments. *** *p* < 0.001 compared to the control group. SLB10, SLB50, and SLB100 represent SLB at concentrations of 10, 50, and 100 μg/mL, respectively.

RAW 264.7 cells play a crucial role in reducing inflammatory responses by clearing pathogens through their phagocytic ability. As shown in Figure 6a, Zymosan (indicated by white arrows) effectively activates the phagocytic function of RAW 264.7 cells, promoting the internalization of Zymosan particles. However, when treated with a specific inhibitor, Zymosan visibly clusters around RAW 264.7 cells without being internalized, indicating that the inhibitor significantly suppresses phagocytic activity.

Interestingly, as illustrated in Figure 6b, low concentrations of SLB enhance the phagocytic ability of RAW 264.7 cells by 3.94% compared to the control group, suggesting that SLB at lower doses may stimulate phagocytic activity. However, with increasing SLB concentrations, phagocytic function declines. When these findings are combined with the observations in Figure 6a, it becomes evident that higher concentrations of SLB significantly reduce the presence of Zymosan under the microscope, indicating that the particles are rapidly cleared by RAW 264.7 cells.

Therefore, the decrease in phagocytic activity observed in the ELISA assay is not due to an inhibition of phagocytic function but rather reflects the accelerated clearance of Zymosan facilitated by higher concentrations of SLB. This suggests that SLB effectively enhances the phagocytic function of RAW 264.7 cells.

### 2.8. Effect of SLB on Cytokine Production in HaCaT Cells Treated with Heat-Inactivated C. acnes

**Figure 7:**
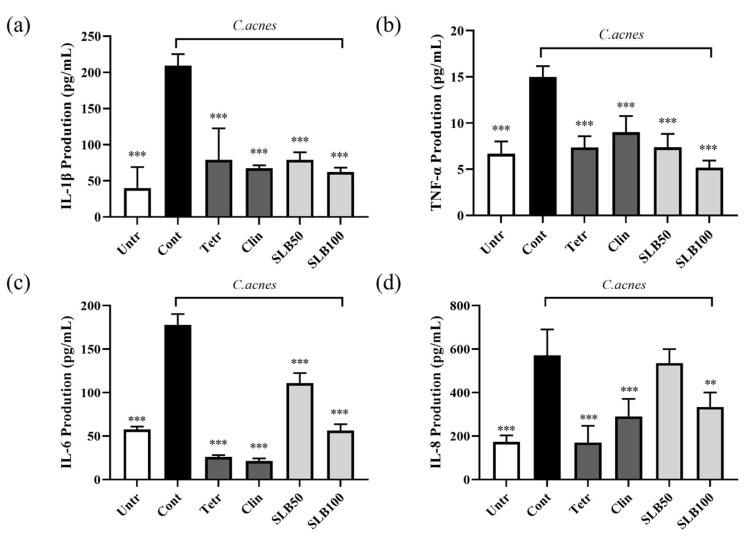
The inhibitory effect of SLB on cytokine production in HaCaT cells induced by heat-inactivated *C. acnes*. ELISA results for cytokines IL-1β (**a**), TNF-α (**b**), IL-6 (**c**), and IL-8 (**d**) in the supernatant. The presentation of the results involves the average ± SD from three independent experiments. ** *p* < 0.01, *** *p* < 0.001 compared to control group. Clin and Tetr refer to clindamycin (10 μg/mL) and tetracycline (10 μg/mL), respectively. SLB50 and SLB100 represent SLB at concentrations of 50 and 100 μg/mL, respectively.

In acne treatment, the production of IL-6 is regulated by IL-1β and TNF-α, which further amplifies the inflammatory cascade [18]. Additionally, IL-8 is induced as part of this process, acting as a potent chemoattractant that recruits neutrophils to the site of inflammation, thereby exacerbating the inflammatory response [19]. As shown in Figure 7, treatment with heat-inactivated *C. acnes* KACC 11946 significantly increases the secretion of IL-1β, TNF-α, IL-6, and IL-8 by 422.93%, 124.38%, 209.08%, and 229.54%, respectively.

SLB effectively suppresses the production of all four cytokines. Although SLB inhibitory effect on IL-6 and IL-8 is less potent than that of the positive controls, clindamycin, and tetracycline, it demonstrates superior inhibition of IL-1β and TNF-α at a concentration of 100 μg/mL compared to 10 μg/mL of clindamycin and tetracycline. These findings suggest that SLB exhibits inhibition of IL-1β and TNF-α comparable to that of antibiotics.

### 2.9. Effect of SLB on MMPs Gene Expression in C. acnes-Treated HaCaT Cells

Under *C. acnes* infection, keratinocytes are induced to secrete large amounts of MMPs [20], significantly accelerating the degradation of the ECM. The degradation of the ECM weakens the structural support of the tight junctions [21], leading to barrier dysfunction and reduced defense capability, which further increases *C. acnes* permeability and creates a vicious cycle.

**Figure 8:**
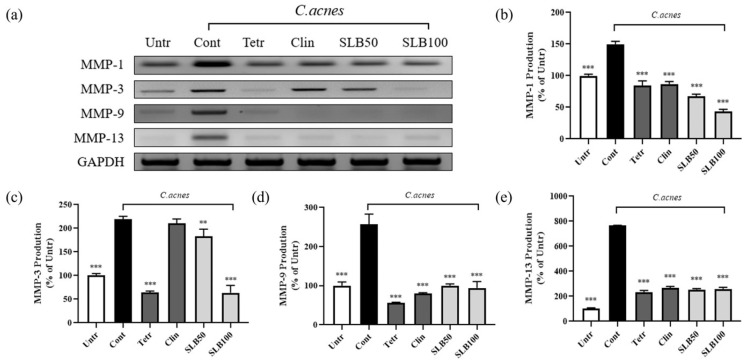
The inhibitory effect of SLB on MMP gene expression in HaCaT cells induced by heat-inactivated *C. acnes*. The RT-PCR results show the inhibitory effect of SLB on MMP gene expression (**a**), with quantified data displayed in histograms for MMP-1 (**b**), MMP-3 (**c**), and MMP-9 (**d**), and MMP-13 (**e**). The presentation of the results involves the average ± SD from three independent experiments. ** *p* < 0.01, *** *p* < 0.001 compared to the control group. Clin and Tetr refer to clindamycin (10 μg/mL) and tetracycline (10 μg/mL), respectively. SLB50 and SLB100 represent SLB at concentrations of 50 and 100 μg/mL, respectively.

Experimental data (Figure 8b–e) show that *C. acnes* treatment significantly upregulated the expression of MMP-1, MMP-3, MMP-9, and MMP-13 by 49.69%, 119.31%, 157.17%, and 665.15%, respectively. This pronounced increase is closely associated with barrier damage and the spread of inflammation. SLB demonstrated a significant inhibitory effect on the expression of MMP-1, MMP-3, MMP-9, and MMP-13, particularly at a concentration of 100 μg/mL, where its inhibition of MMP-1 and MMP-3 was notably superior to that of clindamycin and tetracycline. Interestingly, while clindamycin and tetracycline showed comparable efficacy in suppressing MMP-1, MMP-9, and MMP-13, clindamycin exhibited a marked deficiency in regulating MMP-3, with an inhibition rate of only 4.12%.

## 3. Discussion

Natural plant-based therapies have demonstrated notable advantages over antibiotics in acne treatment, including the ability to circumvent antibiotic resistance, reduced side effects, and multifunctional therapeutic potential [22,23]. These benefits position plant-based treatments as a promising alternative or complementary approach in the field of acne management [24]. In this study, we explored the potential of SLB for anti-acne product development by systematically evaluating its in vitro antibacterial activity, anti-inflammatory effects, and ability to mitigate skin barrier damage induced by *C. acnes*. These findings aim to establish SLB as a viable candidate for innovative and effective acne treatments.

*C. acnes* is an anaerobic Gram-positive bacterium commonly found on the skin, classified into four subtypes based on genomic characteristics: IA, IB, II, and III [25]. Among these, type II has minimal pathogenicity, while type IA is associated with severe inflammatory acne, type IB is linked to mild inflammatory acne, and type III is primarily related to deep follicular infections [26]. When sebaceous glands in hair follicles become blocked, creating an oxygen-deprived and enclosed environment, *C. acne* proliferates rapidly, triggering inflammation and ultimately leading to acne formation [27]. Our results, shown in Figure 2, Figure 3 and Figure 4, demonstrate that SLB not only significantly inhibits the growth of *C. acnes* strains KACC 11946 and KCTC 5012 but also effectively disrupts their biofilm formation. Additionally, as shown in Supplementary Figure S1, SLB treatment exhibited rapid and sustained antimicrobial activity, significantly reducing bacterial viability within 8 h for KACC 11946 and 16 h for KCTC 5012 at MIC. Complete growth suppression was maintained for up to 48 h post treatment, indicating a strong and prolonged bactericidal effect. Notably, KACC 11946 belongs to the IA subtype, while KCTC 5012 is classified as IB, indicating that SLB exhibits inhibitory effects against both the IA and IB subtypes.

Although previous studies have shown that the pathogenicity of different *C. acnes* strains varies, the underlying cause of pathogenicity lies in the production of free fatty acids and other metabolites during the metabolism of sebum by *C. acnes* [28,29]. These metabolites activate Toll-like receptors and the nuclear factor-κB signaling pathway in keratinocytes [30], inducing the release of pro-inflammatory cytokines such as TNF-α, IL-1β, and IL-6 [31]. Our experimental results (Figure 8) show that SLB can effectively suppress the production of pro-inflammatory cytokines in HaCaT cells induced by *C. acnes*. Additionally, these pro-inflammatory cytokines, including IL-8 and CCL2 (monocyte chemoattractant protein-1) [6], can activate endothelial cells in the surrounding follicular tissue, increasing vascular permeability and promoting the migration of immune cells such as macrophages. This process further induces neutrophil infiltration, exacerbates local inflammation, and delays tissue repair. However, Figure 6 and Figure 7 reveal that SLB significantly enhances the phagocytic function of RAW 264.7 cells and reduces the production of NO. This finding suggests that SLB decreases the proportion of M1-type macrophages, alleviating the inflammatory response and ultimately promoting acne recovery.

During the progression of acne inflammation, skin barrier damage is a significant and critical accompanying phenomenon [32]. Studies have shown that inhibiting the excessive expression of MMPs can effectively protect the skin barrier and mitigate tissue damage caused by inflammation [33]. Clinical data indicate high levels of MMP-1 and MMP-13 in the facial sebum of acne patients [34]. Further research has revealed that keratinocytes are a major source of MMPs in acne [35]. Additionally, the protease activity of *C. acnes* can activate protease-activated receptor-2, inducing the expression of various MMPs, including MMP-1, MMP-2, MMP-3, MMP-9, and MMP-13 [20]. Among these, MMP-1 and MMP-13 primarily degrade collagen in the ECM, while MMP-2 and MMP-9 indirectly compromise ECM integrity by degrading hyaluronic acid [36]. These processes not only significantly weaken the mechanical stability of skin tissues but also further disrupt the structure of the basement membrane [37], inhibit the generation of new collagen, reduce tissue repair capacity, and markedly increase the risk of scar formation [38]. Therefore, regulating the activity of MMPs is crucial for protecting the skin barrier, alleviating inflammatory responses, and promoting tissue repair [39]. Experimental results from Figure 8 demonstrate that SLB significantly inhibits *C. acnes*-induced MMP production, further indicating its notable efficacy in intervening in the skin barrier damage caused by *C. acnes*.

## 4. Materials and Methods

### 4.1. SLB Extractions

The dried SLB used in this study was provided by Bozhou Mingjie Biotechnology Co., Ltd. (Bozhou, China). To prepare the extract, 100 g of crushed SLB was soaked in 1 L of 70% (*v*/*v*) ethanol solution and stirred overnight. Afterward, the mixture was passed through 5 µm filter paper (HYUNDAI MICRO, Anseong-si, Republic of Korea) to separate the solids. The filtrate was then concentrated under reduced pressure using a Low-Temperature Circulator CoolAce CCA-1112A (EYELA, Tokyo, Japan) and further processed into a powder through vacuum evaporation, yielding 25.7%. The final SLB extract is now securely stored at Kyung Hee University Global Campus in Yongin, Gyeonggi-do, South Korea.

### 4.2. HPLC

The solution of 1 mg/mL SLB and the standard compounds (baicalin and baicalein) was prepared using 100% methanol as the solvent. Baicalin and baicalein standards were purchased from Tokyo Chemical Industry Co., Ltd. (Tokyo, Japan). HPLC analysis was conducted utilizing the Dionex Chromeleon™ system (Thermo Fisher Scientific, Waltham, MA, USA), which includes a UVD100 detector and a P580 pump. The chromatographic separation was carried out on a Discovery C18 column (25 cm × 4.5 mm, 5 µm; Supelco, Inc., Bellefonte, PA, USA). Additional details regarding the analytical methods can be found in Supplementary Table S1.

### 4.3. Well Diffusion Assay

*C. acnes* strains KACC 11946 and KCTC 5012 were procured from the Korean Agricultural Culture Collection (KACC) and the Korean Collection for Type Cultures (KCTC), respectively. A bacterial suspension (1 × 10^6^ CFU/mL) was spread evenly onto agar plates using sterile cotton swabs. Wells were created on the agar using a puncher, and 100 μL of each sample (SLB, clindamycin, and tetracycline) were added to the wells.

The plates were placed in an MGC AnaeroPack rectangular jar to create anaerobic conditions and incubated at 37 °C for 24 h. The antibacterial effect was assessed by measuring the diameters of the inhibition zones surrounding the wells.

### 4.4. MIC and MBC Assay

The MIC and MBC were determined using the 96-well microdilution method. *C. acnes* strains KACC 11946 and KCTC 5012 were incubated with SLB in a 96-well microplate under anaerobic conditions at 37 °C for 24 h. The absorbance was measured at 600 nm to assess bacterial growth.

### 4.5. Scanning Electron Microscopy

*C. acnes* KCTC 5012, either untreated or exposed to the MIC of the test sample, was subjected to centrifugation at 4000 rpm for 15 min to isolate the bacterial pellet. The collected pellet was fixed in Karnovsky’s fixative at 4 °C for 24 h and subsequently rinsed three times with 0.05 mol/L sodium cacodylate buffer. For post-fixation, the pellet was treated with 1% osmium tetroxide at 4 °C for 1 h, followed by three washes with sterile distilled water.

Bacteria were dehydrated using a graded ethanol series (30–100%, with 100% repeated three times) and treated with 100 μL hexamethyldisilazane for 24 h, after being coated and imaged with an SU8010 scanning electron microscope (Hitachi, Tokyo, Japan).

### 4.6. Biofilm Formation

*C. acnes* strains KACC 11946 and KCTC 5012 were diluted in 0.85% NaCl solution to prepare inocula at 1 × 10^6^ CFU/mL concentrations. Each microplate well was inoculated with the prepared suspension and treated with SLB (0.08–2.5 mg/mL). The plates were maintained at 37 °C for 24 h in an anaerobic environment. After incubation, the supernatant was removed, and each well was washed thrice. The plates were then air-dried. Biofilms formed at the bottom of the wells were stained with 100 μL of 0.01% crystal violet solution. To quantify the biofilm, 33% acetic acid (200 μL) was added to dissolve the biofilm, and absorbance was read at 595 nm.

### 4.7. Cell Culture

The macrophage RAW 264.7 and HaCaT keratinocytes were obtained from the Korean Cell Line Bank (KCLB, Seoul, Republic of Korea). The cells were cultured in DMEM (Gibco, Carlsbad, CA, USA) supplemented with 10% heat-inactivated fetal bovine serum (FBS, Gibco) and penicillin (100 U/mL) at 37 °C in a humidified atmosphere containing 5% CO_2_.

### 4.8. NO Assay

RAW 264.7 cells were plated at a concentration of 1 × 10^6^ cells per well in a 24-well plate and incubated overnight. The cells were pre-treated with SLB, clindamycin, or tetracycline for 1 h, then stimulated with 1 μg/mL LPS overnight. Subsequently, the supernatant (100 μL) was transferred to a 96-well plate. An equal volume of Griess reagent (100 μL) was added and incubated for 10 min. Absorbance at 595 nm was measured to assess nitric oxide production. 

### 4.9. Cytotoxicity

After the NO assay, 100 μL of MTT solution (0.1 mg/mL) was added and incubated overnight. The supernatant was then carefully removed and added 400 μL of DMSO. Absorbance was measured at 595 nm using a microplate reader to assess cell viability.

### 4.10. Phagocytosis Assay

Phagocytic activity was measured using the Cytoselect 96-Well Phagocytosis Assay (Zymosan Substrate) Kit (Cell Biolabs, San Diego, CA, USA) following the manufacturer’s instructions. Briefly, RAW 264.7 cells were seeded at a density of 1 × 10^5^ cells/well in 100 μL of culture medium in a 96-well plate. When cell confluency reached approximately 80%, the cells were pre-treated with SLB, clindamycin, or tetracycline for 24 h. Control wells were treated with 2 μM cytochalasin D for 1 h prior to the assay. The cells were then treated with Zymosan for 2 h as per the kit protocol. Phagocytosis levels were determined by adding the reaction reagent and measuring absorbance at 405 nm.

### 4.11. Enzyme-Linked Immunosorbent Assay

HaCaT keratinocytes were seeded in 24-well plates at a density of 5 × 10^4^ cells/well and incubated for 24 h. They were then pre-treated with SLB, clindamycin, or tetracycline for 1 h. Following the pre-treatment, the cells were stimulated with heat-inactivated *C. acnes* KACC 11946 (1 × 10^7^ CFU/mL) for 24 h. The supernatants were collected and analyzed for cytokine levels. The concentrations of IL-1β, IL-6, TNF-α, and IL-8 were quantified using ELISA kits (R&D Systems Inc., Minneapolis, MN, USA) according to the manufacturer’s instructions.

### 4.12. RT-PCR

After being treated for 24 h, RNA was extracted using TRIZOL reagent (Invitrogen Life Technologies, Carlsbad, CA, USA). For cDNA synthesis, 2 µg of total RNA was reverse transcribed with oligo-dT18 primers. PCR amplification was conducted using PCR premix (Bioneer Co., Daejeon, South Korea). The sequences of the primers are provided in Supplementary Table S2. Each experiment was performed in triplicate with specificity optimized prior to experimentation.

### 4.13. Statistical Analysis

The analysis of the data was performed utilizing GraphPad Prism 8 (GraphPad Software Inc., La Jolla, CA, USA) and ImageJ software (National Institutes of Health, MD, USA). The presentation of the results involves the average ± SD from three independent experiments, subsequently analyzed using analysis of variance (ANOVA). * *p*-value < 0.05, ** *p*-value < 0.01, and *** *p*-value < 0.001.

## 5. Conclusions

This study highlights the significant antibacterial, anti-inflammatory, and skin barrier-protective properties of SLB in the context of acne management. SLB effectively inhibits *C*. *acnes* growth, biofilm formation, and the production of inflammatory cytokines, while also reducing MMPs expression and alleviating skin barrier damage. These findings suggest that SLB is a promising natural therapeutic candidate for the development of innovative and safer anti-acne treatments, providing an effective alternative to traditional antibiotics.

## Figures and Tables

**Table 1:** MIC and MBC analysis of SLB against *C. acnes*.

Strain	MIC (mg/mL)	
SLB	*C. acnes* KACC 11946	*C. acnes* KCTC 5012
	0.04	0.08
	MBC (mg/mL)	
	*C. acnes* KACC 11946	*C. acnes* KCTC 5012
	0.16	0.16

## Data Availability

The data presented in this study are available in this paper.

## Supplementary Materials

The following supporting information can be downloaded at: https://www.mdpi.com/article/10.3390/molecules30030515/s1. Figure S1: Time-kill assay results of (a) *C. acnes* ATCC 11946 and (b) *C. acnes* KCTC 5012 treated with 1× SLB and 1× SLB. The graph represents the bacterial survival rates over time, demonstrating the antimicrobial effects of the treatments; Table S1: HPLC method for the identification of SLB extract; Table S2: Primers used for RT-PCR.
