# Supplementary material for: An In Vitro Study of the Anti-Acne Effects of Scutellaria barbata
Source: Molecules. 2025 Jan 23;30(3):515. doi: 10.3390/molecules30030515 (PMC11819758; doi:10.3390/molecules30030515)
Supplement: Supplementary file 1 [file molecules-30-00515-s001.zip › molecules-3398324-supplementary.pdf]

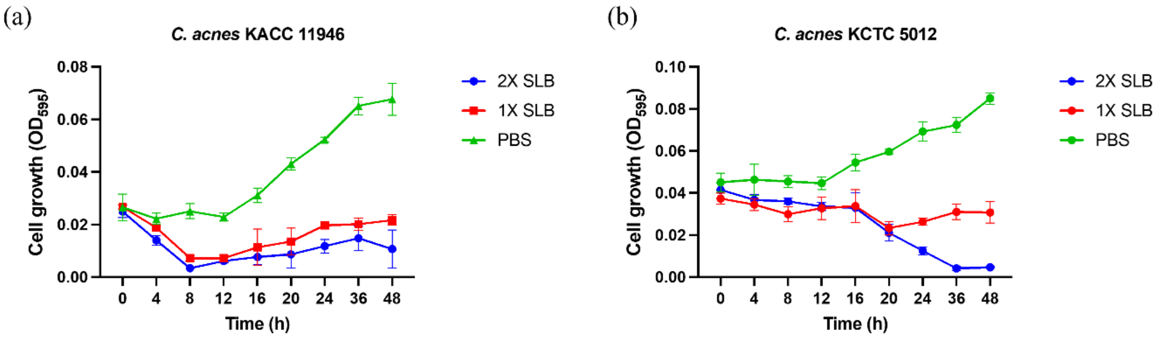

Figure 1. Time-kill assay results of (a) *C. acnes* ATCC 11946 and (b) *C. acnes* KCTC 5012 treated with 1× SLB and 2× SLB. The graph represents the bacterial survival rates over time, demonstrating the antimicrobial effects of the treatments.

Table S1. HPLC method for the identification of SLB extract

**Mobile phase A**	0.1% Formic acid in DW
**Mobile phase B**	Acetonitrile
**Gradient linear**	20% B to 100% B to 20% B
**Retention time**	55min
**UV detection**	270 nm
**Temperature**	25 °C
**Flow-rate**	1 mL/min

Table S2. Primers used for RT-PCR

Primer		Sequence (5’-3’)
Human MMP-1	Sense	TGC GCA CAA ATC CCT TCT AC
	Antisense	TTC AAG CCC ATT TGG CAG TT
Human MMP-2	Sense	TTGACGGTAAGGACGGACTC
	Antisense	ACTTGCAGTACTCCCCATCG
Human MMP-3	Sense	CCC GAG GTT GGA CCT ACA AG
	Antisense	CTT CCC CGT CAC CTC CAA TC
Human MMP-9	Sense	TTGACAGCGACAAGAAGTGG
	Antisense	GCCATTCACGTCGTCCTTAT
Human MMP-13	Sense	AACATCCAAAAACGCCAGAC
	Antisense	GGAAGTTCTGGCCAAAATGA
Human GAPDH	Sense	ACC ACA GTC CAT GCC ATC AC
	Antisense	CCA CCA CCC TGT TGC TGT AG
